# Poly(butylene succinate)/bamboo powder blends as solid-phase carbon source and biofilm carrier for denitrifying biofilters treating wastewater from recirculating aquaculture system

**DOI:** 10.1038/s41598-018-21702-5

**Published:** 2018-02-19

**Authors:** Dezhao Liu, Jiawei Li, Changwei Li, Yale Deng, Zeqing Zhang, Zhangying Ye, Songming Zhu

**Affiliations:** 1 Institute of Agricultural Bio-Environmental Engineering, College of Biosystems Engineering and Food Science, Zhejiang University, Hangzhou, 310058 China; 2 Aquaculture and Fisheries Group, Department of Animal Sciences, Wageningen University, 6708 WD Wageningen, The Netherlands

## Abstract

In this study, Poly(butylene succinate)/bamboo powder (PBS/BP) was newly applied and tested for 8 months as the carbon source in two moving bed reactors for nitrate removal in real RAS wastewater (fresh/sea water), with the purposes of simultaneous reducing the cost of PBS packing and effluent DOC. Fast start-ups were obtained in both reactors, in which high denitrification rates were observed (0.68 ± 0.03 and 0.83 ± 0.11 kg ${{\bf{NO}}}_{{\bf{3}}}^{-}$-N m^−3^ d^−1^ for fresh and sea water, respectively) with no nitrite and low ammonia accumulation. Reduced DOC concentrations in the effluents were also observed compared to pure PBS. The freezing of PBS/BP showed a further slower release of DOC, which might be beneficial to the life of the PBS/BP for the denitrification process, however, microbial activity, especially in high salinity wastewater, was observed to have declined. Illumina sequencing revealed that the autotrophic genus *arcobacter* was discovered first time in solid-phase denitrification system with salinity. Redundancy analysis (RDA) was used to reveal the relationships between environmental factors and the microbial community. In overall, PBS/BP blends were proven to be an economically attractive carbon source for nitrate removal in RAS.

## Introduction

The recirculating aquaculture system (RAS), which is an intensive aquacultural pattern that is an alternative to traditional systems^1^, makes it possible to achieve high fish production, while improving nutrient recycling and diminishing the addition of fresh water^2^; it has thus been hailed as one of the most important future food sources. Nitrifying filters employed in RAS can internally treat water contaminated by dissolved organics and ammonia by oxidizing ammonia to nitrate with nitrite as an intermediate product^3^. Thus, nitrate is often largely accumulated during this process^4^, and the highest concentration of nitrate in RAS can reach 400–500 mg ${{\rm{NO}}}_{3}^{-}$-N L^−1^. Although nitrate is relatively non-toxic to aquatic species, high nitrate concentrations have been observed to be a long-term threat^5^, and a daily water exchange rate (10–20%) is therefore usually needed in order to maintain a relatively low level of nitrate^6^. This not only causes a huge waste of water resources and places a high economic pressure on fish producers but also introduces a big environmental problem by discharging nitrate wastewater. Therefore, the removal of nitrate from RAS has been an urgent task worldwide regarding waste treatment, animal welfare, and environmental sustainability.

Microbial heterotrophic denitrification, using nitrate as the electron acceptor and organic carbon as the electron donor, can transform nitrate to nitrite, nitric oxide, nitrous oxide, and finally nitrogen gas, and it has been proven to be a feasible method to eliminate nitrate from wastewater^7^. Traditionally, different types of liquid organic carbon sources such as methanol, ethanol, or acetic acid were added intentionally to introduce electron donors in such denitrification processes^8,9^. Nevertheless, this solution is sophisticated and it is costly to control the dosage of liquid carbon, the over or under dosing of which would be detrimental to the system stability^10^.

In contrast to liquid carbon sources, denitrification supported by a carbon source of solid organic substances, such as natural materials or biodegradable polymers (BDPs), can avoid the problem of over or under dosing, and it has the benefits of simple process control, less secondary organic pollution and an everlasting supply of carbon^11^. Natural organic substances, such as woodchips (removal efficiency, 60–100%), wheat straws (removal efficiency, 75–90%), corncobs (removal efficiency, 56–90%) and giant reeds (removal efficiency, 30–100%), can all be used as organic carbon sources for denitrification^4,12–15^. However, the heavy color of the effluent and the relatively low denitrification rates inhibited its application for nitrate treatment^16^. BDPs, for instance, polycaprolactone (PCL) polyhydroxybutyrate (PHB) polylactic acid (PLA) and poly(butylene succinate) (PBS), can, on the other hand, slowly release organic carbon compounds, which are first hydrolyzed by extracellular enzymes to their monomer, dimer, trimer, tetramer and other forms and then can be further biodegraded or used directly as electron donors for denitrification as well as carbon sources for living cells^17–22^. BDPs, normally with a high nitrate removal efficiency and with carbon dioxide and water as the final product, have been studied widely, but their shortcomings include high prices and high effluent DOC, which are not beneficial for their extensive application^23^.

Therefore, one of the greatest challenges for solid-phase denitrification is to develop a desirable solid carbon source with low cost, sufficient but not too excessive amounts of surplus electron donors for biological denitrification, and no side effects for effluent water quality. Blending BDPs with a cheap organic material such as bamboo powder or starch was proven to be a promising way to lower their cost while maintaining their good bioavailability for nitrate wastewater treatment^24^. Bamboo, as a clean and cheap material with great tensile and impact strength, has alone been demonstrated to be an acceptable carbon source for nitrate removal in groundwater treatment^25^. Furthermore, PCL/BP blends and PBS, PCL, PLA and PHBV blended with starch have all been proven to be environment-friendly biopolymer blends with relatively low cost^24,26,27^. Chu *et al*.^28^ used PHBV/starch and PHBV/BP as the carbon source and biofilm carrier for the nitrate removal treatment of underground water without external inoculation^28^. Fast start-ups were obtained both in PHBV/Starch and PHBV/BP blend bioreactors, while the PHBV/BP packed reactor exhibited better nitrate removal efficiency (87.4 ± 7.0%), less adverse effects of nitrite accumulation and less DOC release during stable operation. However, these favorable controlled-released carbon-sources (CRCS) with low cost have so far rarely been employed in RAS^21^, whose water characteristics might be distinguished differently from, for instance, underground water by having a higher nitrate concentration and more complex effluent substrate components in RAS than that of groundwater. In addition, DOC is one of the most important factors that affects the denitrification performance, and its release is closely associated with sulfate reduction and the dissimilatory nitrate reduction to ammonium (DNRA) electron competition with denitrification. Zhang *et al*. (2006) reported that the release of DOC was affected by different temperature (60–90 °C) and time treatments^29^, but the effect of low temperature (<0 °C) on the release of DOC was not studied. Therefore, further research of CRCS to determine how to control the release of DOC and improve its life is essential in terms of the economic and environmental sustainability of RAS.

In this study, to test our hypothesis that PBS and BP can act in a mutually complementary manner to both save cost and control the release of DOC, two up-flow fixed bed reactors (with salinities of 0‰ and 25‰) packed with PBS/BP for treating real RAS wastewater were operated for 240 days and evaluated under various operational conditions. The objectives of the study were: 1) to evaluate the performance of the two reactors for nitrate removal, ammonia formation, nitrite accumulation and effluent DOC for RAS wastewater treatment; 2) to analyze the effect of salinity, hydraulic retention time (HRT) and freezing treatment on the denitrification performance and DOC release; 3) to obtain a deeper understanding of the PBS/BP biodegradation characteristics using FTIR and SEM observations; and 4) to identify microbial diversities using Illumina high-throughput sequencing and then to elucidate the correlation between environmental factors and the microbial community using redundancy analysis (RDA).

## Results and Discussion

### The start-up characteristics and denitrification performance

**Figure 1:**
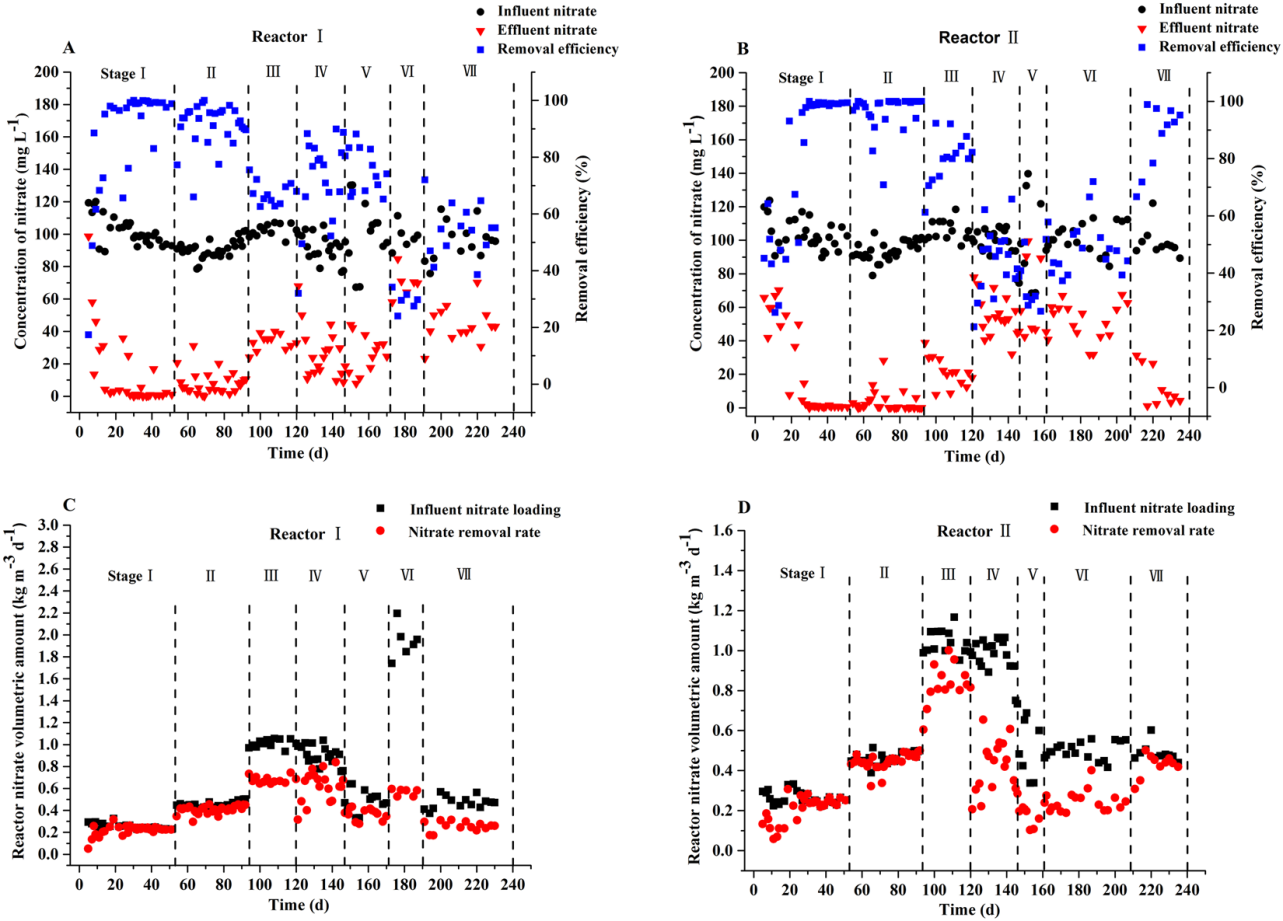
The nitrate removal efficiency (panel A and B) and volumetric removal rate (panel C and D) of the two reactors.

The nitrate removal performance and the volumetric removal rate of nitrate in the two reactors without inoculation are depicted in Fig. 1.

**Figure 2:**
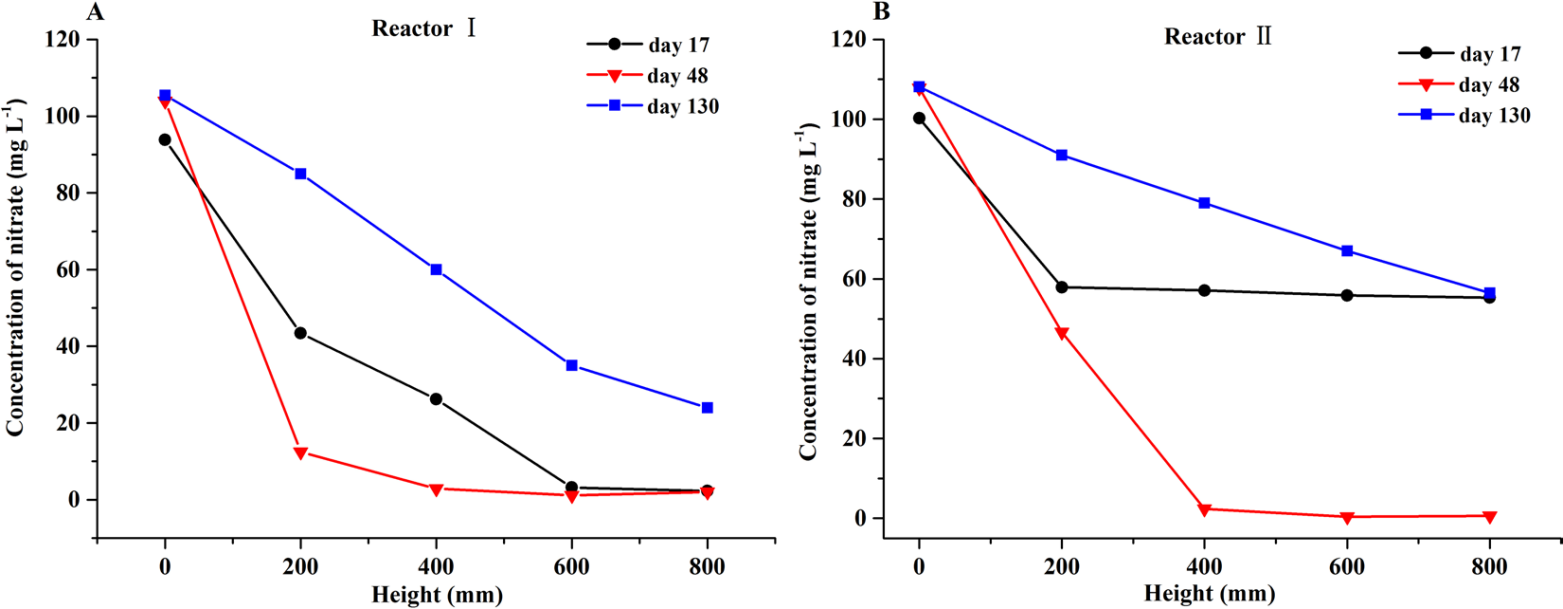
The nitrate concentration profiles along the height of the two reactors at selected operating stages (phase I, on day 17 and 48; phase IV, on day 130).

Stage I comprised the starting operation period and the stable one. It was recently reported that blending PHBV with bamboo powder could significantly shorten the time required for the growth of biofilm compared to a PHBV reactor because bamboo powder could improve bioavailability and favor the utilization and proliferation of microorganisms^28^. In our study, fast start-ups were also obtained in both reactors without external inoculation (to avoid the possible contamination derived from inoculation). Indeed, it only took 8 and 19 days to reach more than 60% of nitrate removal for reactors I and II, respectively. These starting periods were significantly faster when compared to a PBS packed reactor^21,22^, which took 60 days (about 50 mg L^−1^ nitrate; 8 h HRT) and 25 days (50 mg L^−1^ nitrate; 2 h EBRT), respectively, to reach a similar removal efficiency by using activated sludge as the seed for denitrification of synthetic wastewater. Nevertheless, this starting time was slightly longer than that of PHBV/BP blends (ratio 1:1; 6 days) for the treatment of real groundwater with no salinity to reach the same removal efficiency^28^. The higher influent nitrate concentration (approximately 100 mg L^−1^, nearly 6 times that of the PHBV/BP reactor of Chu and Wang, 2016) and shorter HRT (8 hours, half of that of the PHBV/BP reactor of Chu and Wang, 2016) used in this study may be partly responsible for the slower start-up. The start-up time in reactor I was much shorter than that of reactor II, indicating that the growth rate of biofilm in reactor I was significantly faster than that of reactor II and that salinity did not favor the growth of biofilm^30–32^. Further, the nitrate concentration profiles along the two reactors were studied to investigate the distribution of nitrate removal, the growth of biofilm and the denitrification potential, and the results are shown in Fig. 2A,B. On day 17, the nitrate removal (nearly 100%) was distributed throughout reactor I, while for reactor II, the nitrate removal only occurred in the beginning 200 mm of the reactor. This revealed that the low removal efficiency in reactor II was due to the immaturity of biofilm formation, while reactor I had already obtained a successful start-up. On day 48, on the other hand, the nitrate had nearly been removed in the beginning 400 mm of both reactors, indicating a successful start-up of reactor II and the further maturation of the biofilms in both reactors. In addition, during days 1–20, the average effluent DOC concentrations (Fig. 4A,B) in reactors I and II were 157.88 ± 39.21 mg L^−1^ and 126.49 ± 24.12 mg L^−1^, respectively, indicating that there was a sufficient DOC supply in both reactors, which is an important factor to guarantee the success of the reactors. Overall, the salinity, DOC concentration and nitrate loading rate (HRT and nitrate concentration) were likely the main factors associated with the successful and fast start-ups of the two reactors in this study, even though other factors, which were not included here, might also be essential.

During the stable operation period of stage I, the average nitrate removal efficiency was 91.98 ± 12.01% for reactor I, which was close to that observed in stage II (91.08 ± 7.57%), indicating a smooth operation change when the HRT was decreased to half. However, the denitrification rate was improved from 0.23 ± 0.03 to 0.41 ± 0.04 kg ${{\rm{NO}}}_{3}^{-}$-N m^−3^ d^−1^, due to the decreased HRT. Similarly, an average removal efficiency of 94.17 ± 12.38% was obtained for reactor II during the stable period of stage I, with an adjacent averaged removal efficiency in stage II (96.49 ± 6.47%), while the denitrification rate was raised from 0.24 ± 0.03 to 0.45 ± 0.04 kg ${{\rm{NO}}}_{3}^{-}$-N m^−3^ d^−1^.

When the HRT was further changed from 4 hours (stage II) to 2 hours (stage III), the average denitrification rates for reactors I and II were 0.68 ± 0.03 kg ${{\rm{NO}}}_{3}^{-}$-N m^−3^ d^−1^ and 0.83 ± 0.11 kg ${{\rm{NO}}}_{3}^{-}$-N m^−3^ d^−1^, respectively. This performance was comparably better than the performance observed for the denitrification process when PBS was solely used (0.53 ± 0.19 kg ${{\rm{NO}}}_{3}^{-}$-N m^−3^ d^−1^ and 0.66 ± 0.12 kg ${{\rm{NO}}}_{3}^{-}$-N m^−3^ d^−1^ for fresh and sea water, respectively)^21^. Despite the improved denitrification rates, the removal efficiency of nitrate for the two reactors (with averages of 67.42 ± 4.00% and 79.94 ± 9.16% for reactors I and II, respectively) had decreased during this stage, likely due to the increased nitrate loading pressure on the biofilm. Overall, the good performance during the first 3 stages (120 days) of the experiment indicated that the mixing of PBS and BP could achieve higher denitrification rates in both reactors for RAS wastewater treatment. Nevertheless, the effluent DOC was much higher than expected (comparable to our previous hypothesis when half PBS was used), indicating a much higher release of DOC from the PBS/BP than our hypothesis.

**Figure 3:**
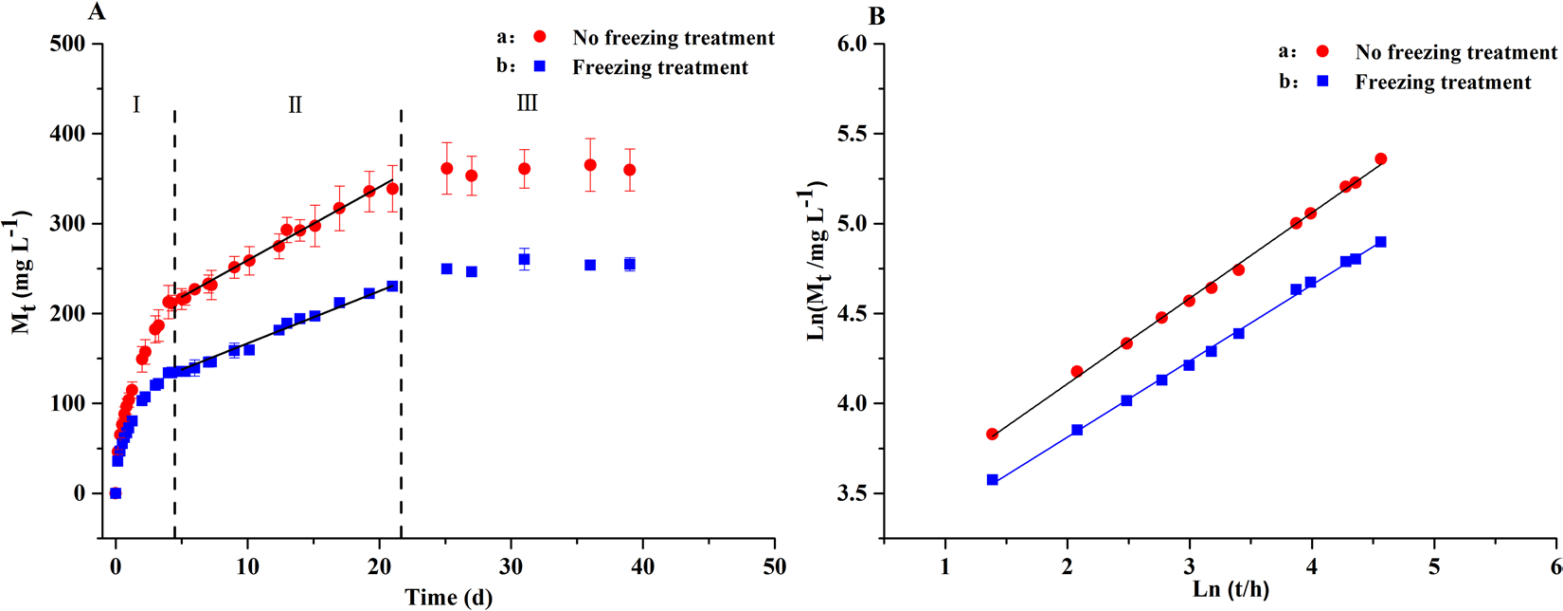
Carbon release process mechanism of PBS/BP polymers by no freezing and freezing treatment (M_t_ was the amount of DOC released at time t. R = 0.9956 and 0.9965 in panel A, R = 0.9991 and 0.9992 in panel B, for no freezing and freezing treatment, respectively).

According to our preliminary experiment (Fig. 3), the rate of DOC released by the PBS/BP blends would be decreased to some extent after freezing at −10 °C. Therefore, to further decrease the effluent DOC concentration as well as to uncover the preservation and recovery characteristics of the carrier/biofilm after exposure to extreme cold conditions, the PBS/BP blends of the two reactors were frozen at −10 °C for one day and then started up again under the HRT of 2 hours (stage IV), the same as that used in stage III. The average denitrification rates in stage IV were 0.61 ± 0.13 kg ${{\rm{NO}}}_{3}^{-}$-N m^−3^ d^−1^ and 0.42 ± 0.13 kg ${{\rm{NO}}}_{3}^{-}$-N m^−3^ d^−1^, respectively, for reactors I and II. The similar denitrification rate for reactor I (before and after freezing) and the much lower denitrification rate for reactor II after restarting indicated that the microorganisms in reactor I had a good toleration ability for freezing and a fast recovery characteristic, while the microorganisms in reactor II were vulnerable to this change of environment. The nitrate concentration profiles along the two reactors on day 130 revealed uniformly distributed biofilms throughout the whole profiles for both reactors, with a significantly lower removal ability of nitrate in reactor II compared to that of reactor I. For both reactors, the removal ability of nitrate was much lower on day 130 (HRT 2 hours) compared with day 48 (HRT 4 hours), while the removal profiles were also significantly different. The longer HRT of 4 hours was therefore tried (stage V) to hopefully improve the performance of the reactors. However, a further decreased nitrate removal efficiency (36.65 ± 21.33%) in reactor II in stage V was observed compared to that in stage IV (44.16 ± 11.14%). This was suspected to be partly caused by the simultaneously decreased DOC released by PBS/BP blends and by the declined activity of the biofilm after the freezing treatment since the abundance values of the denitrifying genera *Arcobacter* in stages IV (12.90%) and V (4.10%) were much lower than those before freezing (average, 20.2%) (Fig. 7B). To study the cause of the decreased performance for reactor II, sodium acetate (approximately 20 mg L^−1^ DOC) was added intentionally in stage VI; afterwards, the salinity was changed from 25‰ to 0‰ in stage VII. A slightly improved nitrate removal efficiency (49.04 ± 9.64%) was achieved in stage VI, while a sharply increased nitrate removal efficiency was obtained in stage VII (87.86 ± 11.46%). Meanwhile, the dominant genera in stage VI for reactor II were found to be *Simplicispira*, *Pseudomonas*, *Denitromonas* and *Thalassospira*, which were nearly the same as those found in stage VII. These results revealed that the high salinity was mainly responsible for the poor performance after reactor II restarted, most likely due to the great restraining effect of salinity on bacteria when the available DOC was relatively insufficient. The result here also indicated that the recovery of a denitrification bioreactor after freezing treatment (for the purpose of slowing the DOC release) under high salinity can take a significantly longer period than the start-up period in the first place. For reactor I, when the HRT was reduced from 4 hours (stage V) to 1 hour (stage VI), the average denitrification rate was further improved (from 0.37 ± 0.05 kg ${{\rm{NO}}}_{3}^{-}$-N m^−3^ d^−1^ to 0.57 ± 0.03 kg ${{\rm{NO}}}_{3}^{-}$-N m^−3^ d^−1^), despite the dramatic decline of the nitrate removal efficiency. The nitrate removal efficiency was, however, not able to reach the same level as found in stage V, when the HRT was increased back to 4 hours in stage VII (75.38 ± 7.68% and 58.02 ± 13.39% in stages V and VII, respectively). Illumina sequencing revealed that the previously dominant genera in stage V, such as *Acidovorax*, *Alicycliphilus* and *Comamonas*, decreased sharply in stage VII (Fig. 7B). These results indicated that a high loading rate of nitrate (in stage VI) likely destroyed the growth of the biofilm, which in return affected the denitrification performance and indicated that the recovery of the biofilm was a long process.

### Carbon release dynamics

The freezing treatment was adopted in a preliminary test as a potential method for slowing down the DOC released by PBS/BP, which may simultaneously further reduce the effluent DOC and prolong the life of the carbon source. To study the effect of freezing treatment on the carbon release, the surface of the PBS/BP blends was embellished by freezing at −10 °C for one day and its carbon release process together with the reference with no freezing treatment are shown in Fig. 3. The carbon release trajectories can be divided into three periods: the rapidly increasing period, the linearly changing period and the slowly close to stable period. During the first several days, the DOC concentrations of all PBS/BP samples increased rapidly, which was likely caused by the weak molecular diffusion resistance because of the low DOC concentration of the solution at the beginning^29^. In addition, the diffluent small molecules on the surface of the materials were easily dissolved in the solution, which favored the rapidly increasing DOC concentration during the first several days. Then, with the increase of the molecular diffusion resistance and the disappearing diffluent substrate on the surface of the materials, the DOC concentrations of the solution increased linearly (k_2_ (mg (L d)^−1^) was the gradient of the linearly changing period). At last, the DOC concentrations gradually became stable, representing a releasing equilibrium.

Many mechanisms and models have been created to describe the carbon release trajectories, and the following equation was used here to describe the kinetics of the carbon release process because of its simple form and fewer parameters^29^:

1 $$\frac{{{\rm{M}}}_{{\rm{t}}}}{{{\rm{M}}}_{\infty }}{=\text{kt}}^{{\rm{n}}}$$

where M_t_ (mg L^−1^) was the amount of DOC released at time t, M_∞_ (mg L^−1^) was the theoretically total amount of DOC released at balance, k was the carbon release coefficient and n was the relative anomalous diffusion as Fickian (n = 0.5), Case II (n = 1.0) and Super Case II (n > 1.0) transport.

Equation (2) was the log-log linear model transformed from equation (1), which was applied to characterize the relationship between the released carbon source (M_t_) and the time (t) in the rapidly increasing period^29^:

2 $${\mathrm{ln}(M}_{{\rm{t}}})={\text{nln}(t)+k}_{1}$$

where ${{\rm{k}}}_{1}$ was the value of the natural logarithm of the product of k and ${M}_{\infty }$.

The results of relevant parameters (presented in Table 1) showed that the carbon release process of PBS/BP blends followed Fickian diffusion, since n was 0.474 and 0.445, and ${{\rm{k}}}_{1}$ was 3.159 and 2.967 for no freezing and freezing treatments, respectively. During the linearly changing period, the gradients of the carbon release trajectories (k_2_) were 8.141 and 5.830 for no freezing and freezing treatments, respectively. Apparently, the values of n, ${{\rm{k}}}_{1}$ and k_2_ decreased after freezing and the lower values of n, ${{\rm{k}}}_{1}$ and k_2_ reflected a slower release of DOC. This indicated that the freezing treatment played a resistant role in the release of DOC. The deterioration of the denitrification performance at low temperatures has been previously reported^33^. In addition, the rate of the linearly changing process was an important parameter to the denitrification because it represented the stable DOC release ability of the materials. Overall, the freezing treatment was favorable for the slow release of the carbon source, but the effect of freezing time on the release of DOC needs to be studied further.

**Table 1:** The parameters of carbon release dynamics.

Treatment methods	M_∞_ (mg L^−1^)	k	n	k_1_	k_2_ (mg (L d) ^−1^)
No freezing treatment	177.897	0.132	0.474	3.159	8.141
Freezing treatment	108.495	0.179	0.445	2.967	5.830

### DOC accumulation

To study the amount of DOC released by PBS/BP, we calculated the values of D_r_ according to equation (3) in the materials and methods of this study; these results are shown in Fig. S1. The mean D_r_ values, which used PBS/BP as the carbon source, were 740 mg d^−1^ and 580 mg d^−1^ for fresh and sea water (reactor I and reactor II), respectively. This result was higher than half of the average D_r_ of PBS mentioned previously, which may be due to the DOC released by the bamboo powder. The HRT and salinity were considered to be the two main factors in our study that affected the value of D_r_. A short HRT of 2 hours obtained higher denitrification rates than a HRT of 4 hours, but the values of its D_r_ were accordingly much higher, which meant that the packing material had a shorter life. This provided a reference for us to comprehensively assess the denitrification rates, the removal efficiency and the life of BDPs when optimizing the operational conditions. In addition, salinity seemed to play a quite different role in the release of DOC in this study than that found in our previous study^21^. In this study, salinity exhibited a significantly restraining role in the release of DOC, while it had an enhancing effect in our previous study. The blending of bamboo powder likely changed the microbial communities in the two reactors, and salinity had a different effect on these microbes, which may account for their diversity. Accordingly, the main genera, *Arcobacter* and *Thalassospira*, in this study might be less active than the genera, *Azoarcus* and *OD1*, in the previous PBS packed reactor with the existence of salinity (25‰).

**Figure 4:**
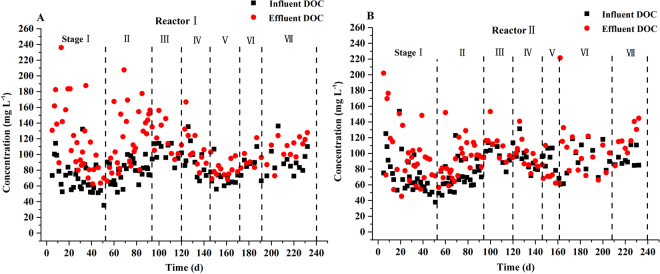
The variation of DOC concentration in the influents and effluents of the two reactors at different operating phases.

The variations of the DOC concentrations in the influents and effluents of the two reactors are depicted in Fig. 4A,B. Overall, the effluent concentrations of the DOC of both PBS/BP packed reactors in this study (with average values of 110.34 ± 33.19 mg L^−1^ and 97.32 ± 29.37 mg L^−1^ for reactor I and reactor II, respectively) were much lower than those found in the PBS packed reactors (with average values of 136.11 ± 49.52 mg L^−1^ and 202.51± 118.90 mg L^−1^ for reactor I and reactor II, respectively) in our previous study. The effluent DOC concentrations were almost identical to the influent DOC values (with average values of 81.44 ± 19.27 mg L^−1^ and 81.20 ± 21.65 mg L^−1^ for reactor I and reactor II, respectively), which reflects the full utilization of the released PBS/BP DOC in consideration of the fact that the influent DOC could not be directly used for denitrification (Fig. S2). When compared with PBS packed reactors, the decrease of the effluent DOC concentration of the PBS/BP packed reactors was much more apparent in sea water, with more than one half decrease. This phenomenon showed that BP is effective in mediating PBS biodegradation. In addition, the freezing treatment seemed to play a restraining role in controlling the effluent quality (with average values of 118.57 ± 7.57 mg L^−1^ and 98.22 ± 29.71 mg L^−1^ for reactor I and reactor II, respectively, before freezing; average values of 98.16 ± 20.26 mg L^−1^ and 95.72 ± 29.08 mg L^−1^ for reactor I and reactor II, respectively, after freezing; and P values of 0.8602 and 0.00107 for reactor I and reactor II, respectively). The results of sea water were not significantly different, possibly due to a more notable effect of salinity than freezing treatment on the release of DOC. On days 1–20, the average effluent DOC concentrations in reactors I and II were 157.88 ± 39.21 mg L^−1^ and 126.49 ± 24.12 mg L^−1^, respectively, and then they decreased slowly. The relatively higher effluent DOC concentrations during the first 20 days were likely caused by the diffluent small molecules on the surface of the materials, which favored the formation and growth of the biofilm and helped to obtain fast start-ups during the starting period. Overall, the mechanisms and dynamics of released DOC require further research, with bacterial quorum sensing as a potential method to investigate how matured carriers influence the microbial colonization and bioactivities of newly replaced carriers.

### Nitrite and TAN accumulation

**Figure 5:**
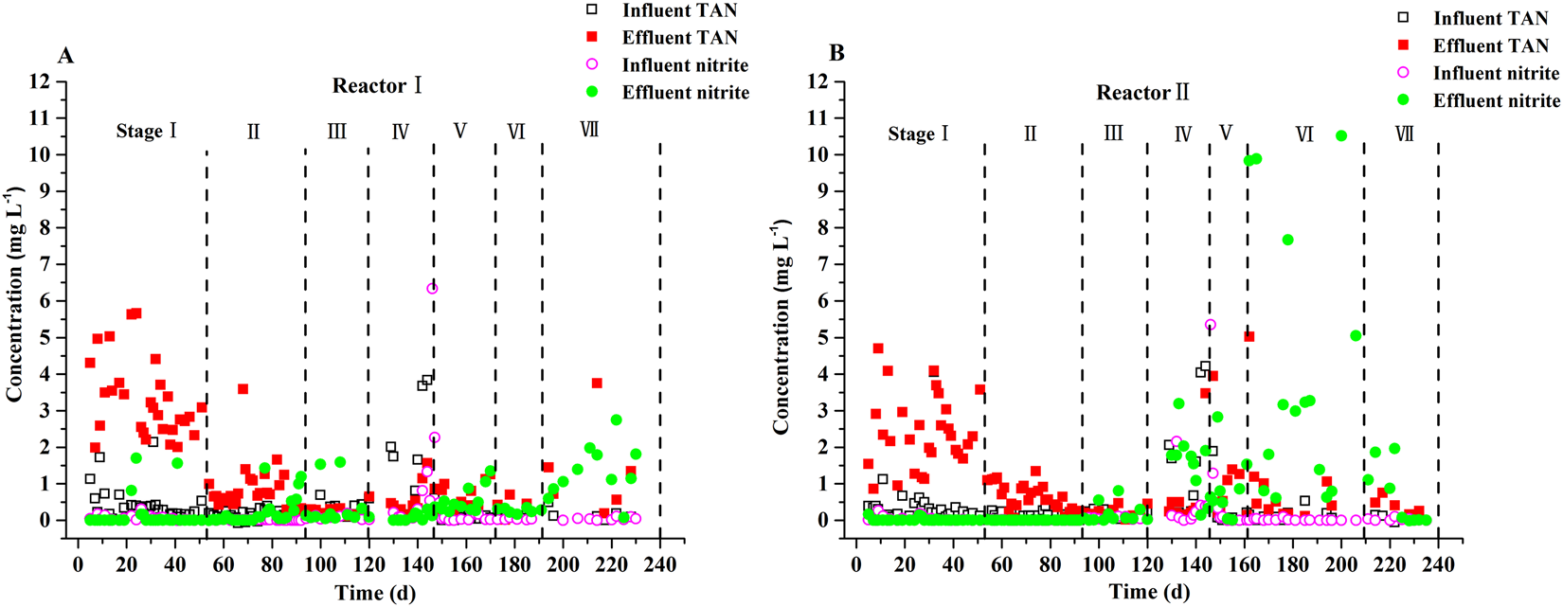
The variations of TAN and nitrite concentrations in the influents and effluents of the two reactors at different operating phases.

During the entire experimental period, TAN accumulation was observed only in Stage I (3.28 ± 1.05 mg L^−1^ and 2.41 ± 0.99 mg L^−1^ for reactor I and II, respectively) (Fig. 5), which was likely caused by DNRA. This result was different from our previous study, when using PBS solely as the carbon source^21^, in which DNRA occurred after day 110 mainly in the sea water reactor (5.35 ± 2.67 mg L^−1^ TAN). Abundant DOC during the period of days 1–52 in this study might account for this difference because a high residual DOC was thought to be a reason for DNRA, as DNRA bacteria have a higher affinity for nitrate compared to denitrifiers when nitrate is limited^34,35^. In this stage, genus *Sulfurospirillum* was also detected in the microbial community, which was affiliated with the DNRA bacteria. Afterwards, due to DOC control, DNRA activity was likely inhibited. In addition, nitrite accumulation occurred after day 120 in reactor II (1.55±0.88 mg L^−1^ in stage IV and 4.64±4.09 mg L^−1^ in stage VI), while there was no obvious nitrite accumulation for reactor I, except during the very last stage. Nitrite accumulation was usually caused by an incomplete denitrification reaction. The weak activity of bacteria in reactor II during days 120–200 was suspected to be the reason for nitrite accumulation, while insufficient DOC was likely to be one of the reasons causing nitrite accumulation in reactor I in the last stage.

### Degradability characterization of PBS/BP by SEM and FTIR

**Figure 6:**
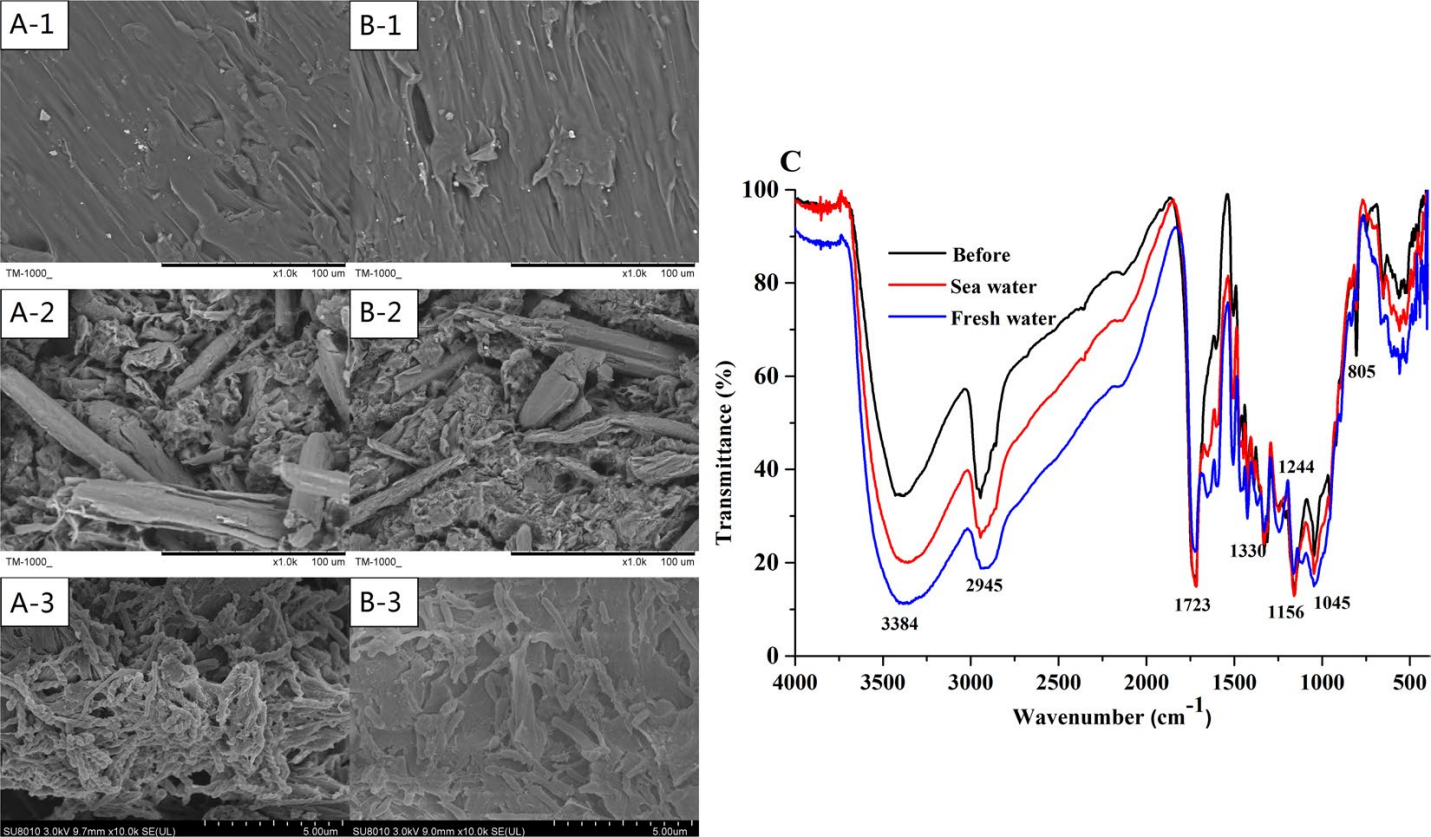
SEM images of fresh (**A**-1, **B**-1) and used BPS/BP blends (**A**-2, **B**-2), and the attached biofilm (**A**-3, **B**-3) of PBS/BP blends. (A: fresh water; B: sea water). The SEM of the attached biofilm was tested at phase VII, on day 207, while the SEM of used carriers was tested right after the whole experiment was finished. FTIR spectroscopy (**C**) of PBS/BP polymers of the two reactors (before used and phase VI, on day 195 for sea and fresh water).

As depicted in Fig. 6, the SEM results of the raw PBS/BP displayed a relatively smooth surface (A-1, B-1), while obvious holes and some dents appeared on the surface of the PBS/BP blends after biodegradation by the bacteria (B-1, B-2). On day 138, the biofilm of reactor I was comparably richer and thicker than that of reactor II (A-3, B-3), verifying the higher denitrification rate of reactor I than that of reactor II during the same stage. Overall, PBS biodegradation and biofilm growth were clearly observed in the SEM micrographs.

The FTIR spectroscopy of the raw and used (day 195) PBS/BP polymers is shown in Fig. 6C. The absorption peaks at 1330 cm^−1^ and 2945 cm^−1^ were ascribed to the symmetric and asymmetric deformational vibrations of the −CH_2_− groups, and the absorption bands at 1723 cm^−1^ and 1156 cm^−1^ were ascribed to the C=O and −C−O−C− stretching of the ester groups (Zhu *et al*.^21^). The positions of characteristic peaks after usage (day 195) had no blue or red shift phenomena, demonstrating that there were no changes in PBS/BP functional groups. Furthermore, the relatively higher density of reactor I than that of reactor II partly explained a faster degradation of PBS/BP polymers in reactor I, which was consistent with the higher release of DOC in reactor I than in reactor II.

### Microbial community analysis

**Figure 7:**
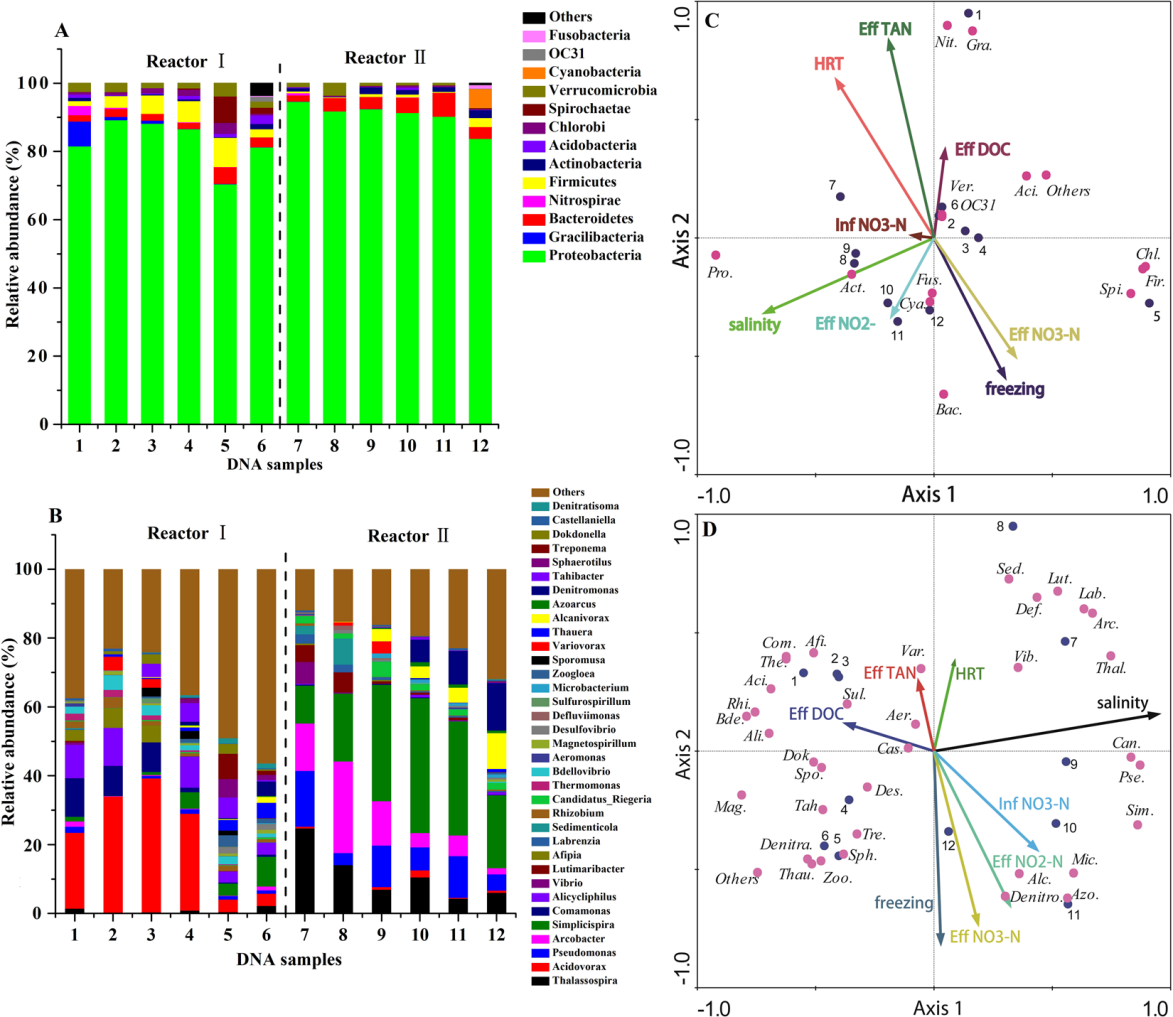
The relative abundances of major bacterial groups at phylum (**A**) and genus (**B**) levels of the two reactors; The redundancy analysis between environmental factors and abundance of microorganism based on phylum (**C**) and genus (**D**) levels of the two reactors. (1–6 were stage I-VII except for Stage III for reactor I; 7–12 were stage I-VII except for Stage III for reactor II; Microbial community was represented by the first three or four letters of bacterium, such as *Proteobacteria* with Pro. for short).

Illumina sequences for 16 S rRNA genes were carried out to reveal the microbial community structures of the two reactors, and the relative abundances of the main phyla (sequencing percentage >1% in at least one sample) are depicted in Fig. 7A. According to the result of the main phyla, the most three dominant microbes were the *Proteobacteria* (average 83.0 ± 7.0%), *Firmicutes* (average 4.6 ± 2.7%) and *Bacteroidetes* (average 2.6 ± 1.2%) for reactor I, and the *Proteobacteria* (average 92.2 ± 1.6%), *Bacteroidetes* (average 4.1 ± 1.9%) and *Actinobacteria* (average 1.1 ± 0.7%) for reactor II. This observation was generally consistent with previous studies, in which PBS^21^ or PHBV/BP^28^ was used as the carbon sources and biofilm carriers in denitrification systems. In fact, the Gram-negative *Proteobacteria* included a wide variety of both aerobic and anaerobic bacteria, which could degrade a broad spectrum of organic substances, as was previously reported^36^. It was also reported in a recent study that the anaerobes *Bacteroidetes* could breakdown macromolecular substances, such as fiber, protein, starch, and bamboo powder, in a fermentation system^28^.

Figure 7B depicts the relative abundances of the main genera of the two biofilm samples. For reactor I, *Acidovorax* (average 25.3 ± 13.7%) was the most dominant, followed by *Alicycliphilus* (average 8.2 ± 3.5%) and *Comamonas* (average 6.0 ± 4.9%) in the first five stages. Here, *Acidovorax* and *Comamonas* likely played the role of partial biodegradation^21^ while *Alicycliphilus* was likely to be the dominant denitrifier in PLA/starch blends^9^, which was consistent with the conclusion that the bacterial community was determined by different carbon sources^4^. In stage VI, *Acidovorax* (3.7%), *Alicycliphilus* (3.2%) and *Comamonas* (3.0%) were all found to have decreased dramatically, which accounted for the low denitrification rate in this period. For reactor II, the most four dominant genera were *Simplicispira* (average 27.4 ± 11.7%), *Arcobacter* (average 12.7 ± 8.8%), *Pseudomonas* (average 10.1 ± 5.0%) and *Thalassospira* (average 12.1 ± 7.9%) during all stages except the last one. Genera *Arcobacter* was mixotrophic, with the ability to reduce nitrate to nitrite^37^. The relatively low abundance of *Arcobacter* in stages IV (4.1%) and V (6.0%) might account for the poor denitrification performance. Other denitrifying genera, such as *Pseudomonas*, *Afipia*, *Rhizobium* and *Thermomonas*, were detected in the wastewater treatment system^21,38^. It is noteworthy that no significant microbial community shift was detected, although the nitrate removal was improved significantly, after the salinity was reduced from 25‰ to 0‰ in reactor II at the beginning of the last stage. This revealed that early microbiota formation likely has an “inpainting” effect on later operations, even though environmental conditions were changed. This effect leads us to hypothesize that when changing salinity from 0‰ to 25‰, microbiota may likely survive and show a high performance for a marine denitrification reactor. However, this hypothesis requires further investigation and validation in the future. Overall, the different types of carbon sources affected the diversity and changes in the abundance and species of denitrifying microbial communities, which played a determinant role in the denitrification performance.

### Relationships between environmental factors and the microbial community

RDA was used to reveal the relationships between environmental factors and the microbial community, and the results are shown in Fig. 7C,D. The freezing treatment was positively correlated with effluent nitrate and effluent nitrite, but negatively correlated with effluent DOC and effluent TAN. This explains why a declined denitrification performance and nitrite accumulation were obtained from the sea water reactor and why salinity had a significantly restraining effect on the release of DOC for reactor II after freezing. The effluent nitrate and effluent nitrite were negatively correlated with the HRT and effluent DOC, which suggested that by raising the HRT, the removal efficiency of nitrate could be improved and the nitrite accumulation could possibly be controlled. This could, however, cause an increase in the effluent DOC at the same time. The effluent TAN had positive correlations with HRT, salinity and effluent DOC, indicating that high effluent DOC accounted for the TAN accumulation and that the TAN accumulation could possibly be controlled by reducing the HRT or the freezing treatment, which, however, likely increased the effluent nitrate. On the other hand, salinity had a negative correlation with the effluent DOC and a positive correlation with effluent nitrite, and the majority of bacteria seemed to have a “rejecting” trend to the salinity except for the phyla *Proteobacteri*a and *Actinobacteria* and genera *Thalassospira*, *Candidatus_Riegeria*, S*implicispira*, *Pseudomonas*, *Arcobacter*, *Labrenzia*, *Lutimaribacter*, *Microbacterium*, etc. Effluent TAN was positively correlated with the phyla *Nitrospirae*, *Gracilibacteria* and genera *Sulfurospirillum*, *Aeromonas*, *Variovorax*, which were suspected to play roles in the DNRA process. Further, effluent nitrite had positive correlations with the phyla *Cyanobacteria* and *Fusobacteria* as well as genera *Denitromonas*, *Alcanivorax*, *Azoarcus* and *Microbacterium*, while effluent nitrate clearly had a negative correlation with the genus *Variovorax*, which was suspected to be an important species for denitrification. Furthermore, effluent DOC had clear correlations with a vast majority of bacterium, such as genera *Rhizobium*, *Acidovorax*, *Bdellovibrio*, *Thermomonas*, *Comamonas*, *Alicycliphilus*, *Afipia*, etc. In conclusion, the relationships between environmental factors and microbial community were well displayed by the RDA; however, further studies are needed to assess the relationships between DNRA bacteria, denitrification bacteria and environmental factors. Further research is also needed to determine how salinity influences bamboo power degradation and utilization and to assess the relationship between salinity and the microbial community.

### Cost evaluation

The cost of nitrogen removal and the denitrification rate of PBS/BP are presented in Table 2, which have been compared with other denitrification solid carbon sources, such as PBS, PHBV/BP and PCL. The unit material prices of the substrate materials were based on the Chinese market in September 2017. Although the denitrification rate and consumption rate of PBS/BP were nearly the same as those of PBS, the nitrogen removal cost of PBS/BP was much lower than that of PBS due to the low price of bamboo powder. When compared with PHBV/BP blends, the denitrification rate of PBS/BP was five times higher, despite its slightly higher price. Furthermore, the nitrogen removal cost of PBS/BP was one-fifth that of PCL, although the denitrification rate of PBS/BP was one-fourth that of the PCL denitrification rate. Overall, the PBS/BP mixture was more economical than other biopolymers, with a relatively higher denitrification performance.

**Table 2:** Estimated cost of carbon source for nitrate removal.

Substance material	Denitrification rate (g N/(L d))	Consumption of substrate (kg/kg N)	Price (CNY/kg)	Nitrogen removal costs (CNY/kg N)	Reference
PBS/BP	0.63–0.8	2.46	12.8	31.49	This study
PBS	0.53–0.66	2.11	24.8	52.33	(Zhu *et al*.^21^)
PHBV/BP	0.13	1.69–1.86	15.5	26.2–28.8	(Chu and Wang, 2016)
PCL	2.65	2.34	68	159.1	(Li *et al*.^33^)

## Materials and Methods

### Reactors and biodegradable PBS/BP materials

The schematic of the two up-flow-fixed bed PBS/BP denitrification reactors was the same as that used in our previous study^21^. The reactors, placed in a temperature-controlled laboratory, were both 1000 mm in height with a 60-mm inner diameter. The PBS and BP granules, purchased from the Anqing He Xing Chemical Corporation Limited and the Institute of Anji Bamboo Business of Zhejiang University, respectively, were blended as described below with a packed height in the reactors of approximately 900 mm.

The PBS/BP (1:1 blending) biopolymers were made into cylindrical granules with an average size of 10 mm × 10 mm (diameter × height) using a mixing roll (HL-200, Scientific and Educational Instrument Factory of Jilin University). The porosity and the density (25 °C) of the PBS/BP granules were measured to be 45.1% and 1.06 kg L^−1^ at the beginning of our experiment. The 1:1 blending of PBS and BP was chosen according to the estimation of the released DOC from the PBS based on our previous study, as stated below.

According to the results of our previous study^21^, the amount of released DOC can be estimated by the following mass balance equation:

3 $${{\rm{D}}}_{{\rm{r}}}={{\rm{D}}}_{{\rm{ef}}}+{{\rm{D}}}_{{\rm{N}}}+{{\rm{D}}}_{{\rm{e}}}-{{\rm{D}}}_{{\rm{in}}}$$

where D_r_ (mg) was the amount of DOC released by BDPs, D_in_ (mg) and D_ef_ (mg) were the amounts of influent and effluent DOC, respectively, D_N_ was the amount of DOC consumed by denitrification, and D_e_ was the amount of DOC consumed by microbes or other mechanisms.

Accordingly, the mean D_r_ values were estimated to be 750 mg d^−1^ and 940 mg d^−1^ for the freshwater and seawater reactors, respectively, according to the previous study (Fig. S1), in which PBS was used as the sole carbon source^21^. The effluent DOC could in theory be reduced to nearly zero, if the released DOC was decreased to half, by assuming D_in_ and D_e_ in the equation above remained unchanged. Following this idea, we blended PBS with bamboo powder in a proportion of 1:1.

### Experimental setup and procedures

The different stages and operational conditions during the whole experiment are depicted in Table 3. During the overall experiment, real RAS wastewater was used and a moderate KNO_3_ solution was added to maintain a nitrate level of 80–120 mg ${{\rm{NO}}}_{3}^{-}$-N L^−1^. The tilapia RAS consisted of two tanks (1000 L) and one MBBR bio-filter that was relatively stably operated in our laboratory for more than one year. For the first three stages, PBS/BP blends with no treatment were used as the carbon source for each of the two reactors. To further decrease the effluent DOC concentration, as well as to uncover the preservation and recovery characteristics of the carrier/biofilm after exposure to extreme cold conditions, the PBS/BP blends were taken from both reactors after the third stage and then treated by freezing at –10 °C for one day before being returned to the reactors. For the following stages (IV–VII), the experiment was operated with PBS/BP blends treated by freezing for the two reactors. The two reactors, operated in parallel, were started up without inoculation at an HRT of 8 hours in stage I, which was then adjusted, depending on the effluent nitrate, in the following stages, as shown in Table 3. To study the cause of the decreased performance after freezing treatment for reactor II, sodium acetate (approximately 20 mg L^−1^ DOC) was added intentionally in stage VI; afterwards, the salinity was changed from 25‰ to 0‰ in stage VII. The artificial sea salt (Blue Starfish Salt Product Co., Ltd., Zhejiang, China) was used to keep the salinity at 25‰ for reactor II during all of the stages except the last one, while reactor I was continuously operated at 0‰. In addition, DNA samples were also collected from two reactors in specific stages to evaluate variations in the bacterial community.

**Table 3:** The different operated conditions of the two PBS/BP reactors (Number 1–12 were sampled from the middle of the two reactors).

		Stage I	Stage II	Stage III	Stage IV	Stage V	Stage VI	Stage VII
Reactor I	**Operation conditions and influent characteristics**							
	Operated time (d)	1–53	54–93	94–120	121–146	147–172	173–190	191–235
	Hydraulic retention time (h)	8	4	2	2	4	1	4
	Salinity (‰)	0	0	0	0	0	0	0
	Influent loading rate (kg ${{\rm{NO}}}_{3}^{-}$-N/m^3^**·**d)	0.25 ± 0.02	0.45 ± 0.03	1.01 ± 0.04	0.90 ± 0.09	0.50 ± 0.1	1.94 ± 0.15	0.47 ± 0.05
	${{\rm{NO}}}_{3}^{-}$-N (mg L^−1^)	100.85 ± 8.07	90.77 ± 5.64	102.47 ± 3.83	91.49 ± 8.98	100.5 ± 19.75	98.40 ± 7.76	95.35 ± 11.03
	${{\rm{NO}}}_{2}^{-}$-N (mg L^−1^)	0.06 ± 0.07	0.01 ± 0.01	0.08 ± 0.05	0.90 ± 1.84	0.27 ± 0.61	0.03 ± 0.02	0.40 ± 1.39
	TAN (mg L^−1^)	0.44 ± 0.48	0.14 ± 0.11	0.33 ± 0.18	1.47 ± 1.39	0.12 ± 0.16	0.16 ± 0.09	0.18 ± 0.16
	DOC	71.29 ± 21.16	78.79 ± 13.24	102.77 ± 10.65	90.78 ± 10.81	70.77 ± 12.11	89.8 ± 19.84	90.12 ± 17.94
	**DNA sampling**							
	Sample time (d)	43	93		140	161	187	228
	Number	1	2		3	4	5	6
Reactor II	**Operation conditions and influent characteristics**							
	Operated time (d)	1–53	54–93	94–120	121–146	147–161	162–210	211–235
	Hydraulic retention time (h)	8	4	2	2	4	4	4
	Salinity (‰)	25	25	25	25	25	25	0
	Influent loading rate (kg ${{\rm{NO}}}_{3}^{-}$-N/m^3^**·**d)	0.26 ± 0.03	0.46 ± 0.03	1.04 ± 0.06	0.97 ± 0.10	0.50 ± 0.04	0.50 ± 0.04	0.49 ± 0.04
	${{\rm{NO}}}_{3}^{-}$-N (mg L^−1^)	102.39 ± 7.6	93.83 ± 5.80	105.54 ± 6.55	98.04 ± 9.98	101.16 ± 8.74	101.73 ± 8.83	98.77 ± 8.93
	${{\rm{NO}}}_{2}^{-}$-N (mg L^−1^)	0.04 ± 0.06	0.00 ± 0.01	0.06 ± 0.03	0.40 ± 0.64	0.23 ± 0.44	0.01 ± 0.03	0.02 ± 0.03
	TAN (mg L^−1^)	0.42 ± 0.73	0.10 ± 0.10	0.16 ± 0.1	1.51 ± 1.55	0.29 ± 0.65	0.13 ± 0.15	0.10 ± 0.06
	DOC	71.59 ± 24.74	72.09 ± 16.98	100.1 ± 11.86	93.13 ± 12.37	76.7 ± 20.63	108.41 ± 21.3	93.19 ± 10.48
	**DNA sampling**							
	Sample time (d)	43	93		140	161	187	228
	Number	7	8		9	10	11	12

Furthermore, the nitrate concentration profiles along the depths of two reactors were determined to investigate the distribution of the nitrate removal at selected operating stages (phase I, on days 17 and 48; phase IV, on day 130). In addition, a preliminary test (as a potential method for slowing down the release of DOC by the freezing treatment of PBS/BP) was adopted in an incubator at 19 °C. The surface of the PBS/BP blends was also embellished by freezing at −10 °C for one day. The DOC concentration was measured every 2–4 hours in the first two days and later every 3–5 days.

The denitrification rate and nitrate removal efficiency of the reactors were calculated according to the following equations:

4 $${{\rm{DEN}}}_{{\rm{r}}}=0{\rm{.24Q}}({{\rm{C}}}_{{\rm{in}}}-{{\rm{C}}}_{{\rm{ef}}})/({\rm{\eta }}V)$$

5 $${{\rm{R}}}_{{\rm{e}}}=({{\rm{C}}}_{{\rm{in}}}-{{\rm{C}}}_{{\rm{ef}}})/{{\rm{C}}}_{{\rm{in}}}\times 100 \% $$

where DENr (kg ${{\rm{NO}}}_{3}^{-}$-N m^−3^ d^−1^) and ${{\rm{R}}}_{{\rm{e}}}$ (%) were the denitrification rate and nitrate removal efficiency, respectively. C_in_ (mg L^−1^) and C_ef_ (mg L^−1^) were the concentrations of ${{\rm{NO}}}_{3}^{-}$-N in the influent and effluent, respectively. Q (L h^−1^) was the flow rate, V (L) was the bulk volume of the PBS/BP, and η was the porosity of the PBS/BP.

### Analytical methods

All the samples were filtered using a 0.45-µm filter membrane before analysis. ${{\rm{NO}}}_{3}^{-}$-N, ${{\rm{NO}}}_{2}^{-}$-N and total ammonia nitrogen (TAN) concentrations were analyzed according to the Chinese SEPA standard methods (SEPA, 2002). DOC was measured using a TOC analyzer (Multi N/C 2100, Analytik Jena, Germany). The structures of the PBS/BP granules and the attached biofilm were observed using a scanning electron microscope (SEM) (TM-1000 and SU8010, Hitachi High-Technologies Corporation, Japan) on days 0, 207, and 240. A Fourier transform infrared (FTIR) spectrometer (Avatar 370, Thermo Nicolet, USA) was used to characterize the changes of the major functional groups of the PBS/BP granules before and after use.

### Biofilm sampling, DNA extraction and PCR amplification

The method of biofilm sampling, DNA extraction and PCR amplification was the same as those used in our previous study^21^. The attached biofilms of different periods were initially extracted from the PBS/BP granules through ultrasonic treatment (SK3200HP, Kedao Ultrasonic Instrument Co., Ltd., China); they were then shaken with a vortex shaker (WH-861, Hualida laboratory instrument Co., Ltd., China) and finally filtered through a 0.22-µm sterile membrane. The extracted biofilm samples were further used for genomic DNA extraction using a FastDNA^®^ Spin Kit for Soil (MP Biomedicals, CA, USA). The PCR amplification of the 16 S rRNA (V3–V4 hypervariable regions) genes from the extracted DNA was operated using the primers 341 F (5′-CCTACGGGNGGCWGCAG-3′) and 805 R (5′GACTACHVGGGTATCTAATCC-3′), as previously described^39^. The PCR amplification program included an initial denaturation step of 3 min at 94 °C, followed by 5 cycles (94 °C for 30 s, 45 °C for 20 s, 65 °C for 30 s) and 20 cycles (94 °C for 20 s, 55 °C for 20 s, 72 °C for 30 s), with a final extension step of 5 min at 72 °C. The PCR mixture consisted of 10 ng DNA template, 1.0 µl primer mix (50 µM), 0.5 µl dNTP mixture (10 mM each), 5.0 µl 10× PCR buffer (2.5 mM), 0.5 µl Platinum Taq (5 U µl^−1^) and sufficient RNase-free water to bring the mixture up to a volume of 50 µl.

### Illumina high-throughput sequencing and bioinformatics analysis

The PCR amplifications were first extracted from 2% agarose gels and then purified using Agencourt^®^ AMPure XP Beads (A63881, Beckman, USA). After this, the quality of the gene library was prepared using a Qubit^®^ 2.0 Fluorometer (Q32866, Invitrogen, USA) and an Agilent 2100 Bioanalyzer (G2939AA, Agilent, USA). At last, the qualified amplifications were run on an Illumina MiSeq platform at the Zhejiang Institute of Microbiology (Hangzhou, Zhejiang, China) according to the standard protocols and software (Data collection software, Illumina).

RDA was applied using Canoco 4.5 to analyze the relationships between environmental factors and the microbial community. The significance of the correlation between environmental factors and the microbial community depended upon the length of the arrows; the longer the arrow, the stronger the correlation^40^. The vector of the relationships (positively proportional or negatively proportional) was interpreted based on the position of the factors or bacterium in terms of the arrow ends. If the factors or bacterium were located close to the end of the arrows, the association was positively correlated; if they were on the opposite site, then the correlation was negative.

## Electronic supplementary material

Supplementary material
