# Supplementary material for: Poly(butylene succinate)/bamboo powder blends as solid-phase carbon source and biofilm carrier for denitrifying biofilters treating wastewater from recirculating aquaculture system
Source: Sci Rep. 2018 Feb 19;8:3289. doi: 10.1038/s41598-018-21702-5 (PMC5818489; doi:10.1038/s41598-018-21702-5)
Supplement: Supplementary file 1 — Supplementary material [file 41598_2018_21702_MOESM1_ESM.pdf]

Poly(butylene succinate)/bamboo powder blends as solid-phase carbon source and biofilm carrier for denitrifying biofilters treating wastewater from recirculating aquaculture system

Dezhao Liu [1], Jiawei Li [1], Changwei Li [1], Yale Deng [2], Zeqing Zhang [1], Zhangying Ye [1, *], Songming Zhu [1]

[1]*Institute of Agricultural Bio-Environmental Engineering, College of Biosystems Engineering and Food Science, Zhejiang University, Hangzhou 310058, China*

[2]*Aquaculture and Fisheries Group, Department of Animal Sciences, Wageningen University, 6708 WD Wageningen, The Netherlands*

[*]Corresponding author: Zhangying Ye.
E-mail address: yzyzju@zju.edu.cn.
Telephone number: 008657188982491.

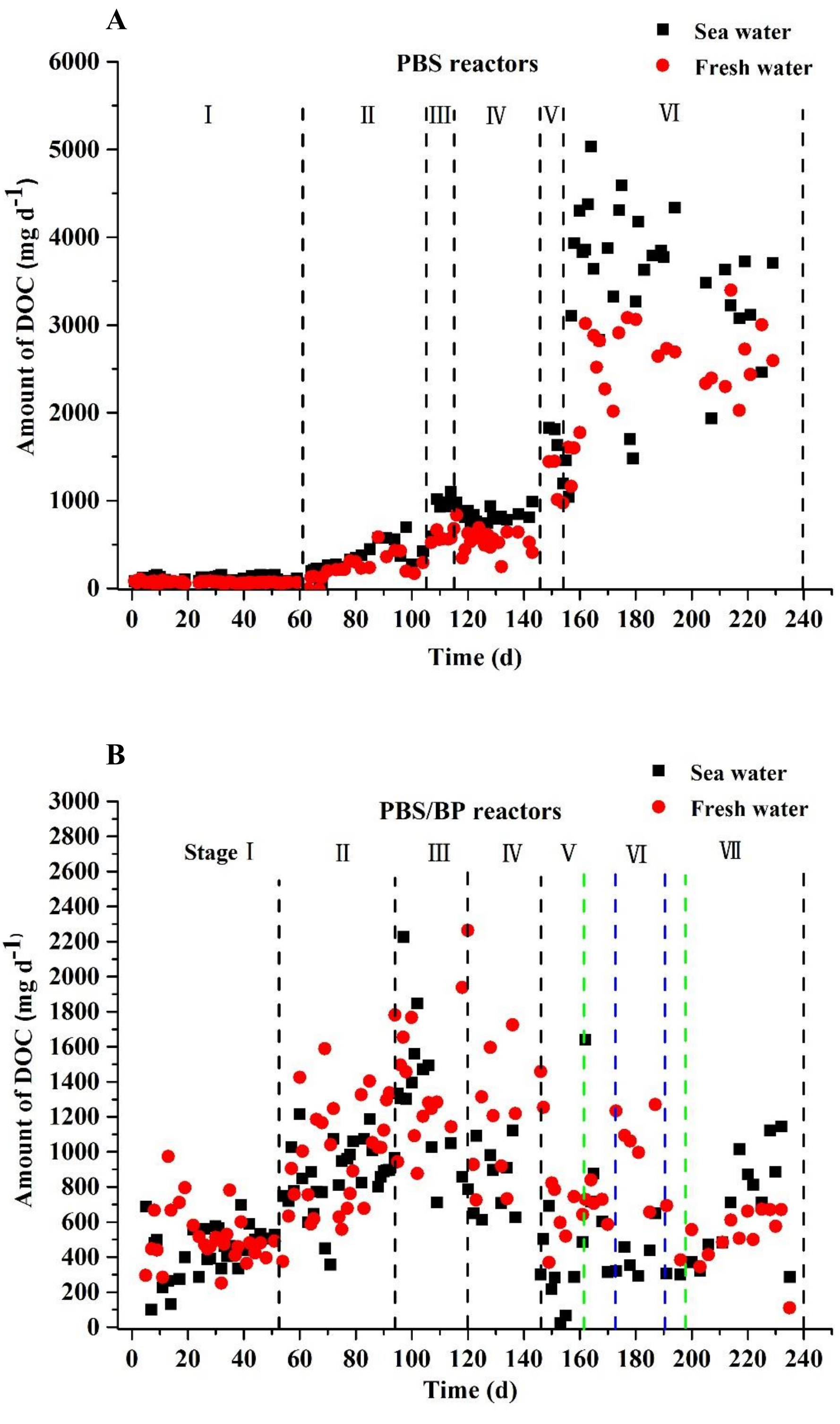

Fig. S1. The daily variation of released DOC (mg d^{-1}) by PBS (panel A) and PBS/BP blends (panel B).

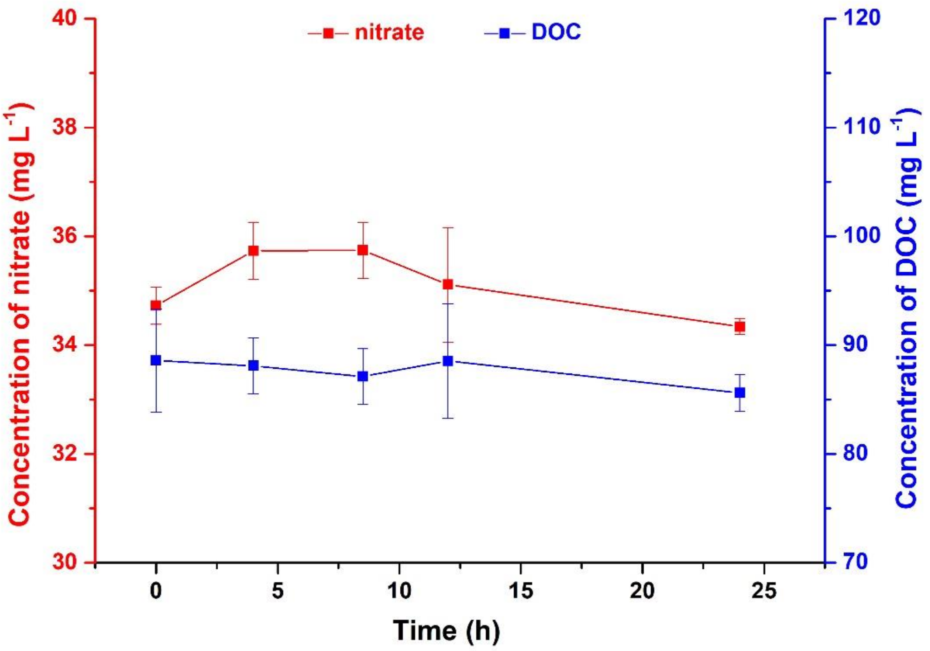

Fig. S2. The variation of nitrate concentration during 24 hours in influent.
